# Do Uric Acid Deposits in Zooxanthellae Function as Eye-Spots?

**DOI:** 10.1371/journal.pone.0006303

**Published:** 2009-07-17

**Authors:** Hiroshi Yamashita, Atsushi Kobiyama, Kazuhiko Koike

**Affiliations:** 1 Graduate School of Biosphere Science, Hiroshima University, Hiroshima, Japan; 2 School of Marine Biosciences, Kitasato University, Ofunato, Japan; Northeastern University, United States of America

## Abstract

The symbiosis between zooxanthellae (dinoflagellate genus *Symbiodinium*) and corals is a fundamental basis of tropical marine ecosystems. However the physiological interactions of the hosts and symbionts are poorly understood. Recently, intracellular crystalline deposits in *Symbiodinium* were revealed to be uric acid functioning for nutrient storage. This is the first exploration of these enigmatic crystalline materials that had previously been misidentified as oxalic acid, providing new insights into the nutritional strategies of *Symbiodinium* in oligotrophic tropical waters. However, we believe these deposits also function as eye-spots on the basis of light and electron microscopic observations of motile cells of cultured *Symbiodinium*. The cells possessed crystalline deposit clusters in rows with each row 100–150 nm thick corresponding to 1/4 the wavelength of light and making them suitable for maximum wave interference and reflection of light. Crystalline clusters in cells observed with a light microscope strongly refracted and polarized light, and reflected or absorbed short wavelength light. The facts that purines, including uric acid, have been identified as the main constituents of light reflectors in many organisms, and that the photoreceptor protein, opsin, was detected in our *Symbiodinium* strain, support the idea that uric acid deposits in *Symbiodinium* motile cells may function as a component of an eye-spot.

## Introduction

Symbioses between zooxanthellae (unicellular algae in the dinoflagellate genus *Symbiodinium*) and marine invertebrates including corals are common in shallow tropical marine environments. *Symbiodinium* contributes to the host's nutrition by translocating photosynthetic products, thus enabling the host to thrive in oligotrophic seas [1]. The loss of *Symbiodinium* from corals, frequently referred to as “coral bleaching”, sometimes results in the mass mortality of coral, and has occurred with increased frequency over the past two decades [2]. Coral bleaching events have become a great concern not only with regard to the conservation of tropical and subtropical coral reef ecosystems, but also as a possible indication of global warming.

Knowledge of the physiological interactions between zooxanthellae and the host animals is limited. *Symbiodinium* cells can live independently outside the host animals, and have been found in the water column [3], [4], sands [4], [5], and on macroalgal surfaces [6]. The presence of free-living population pool of *Symbiodinium* is necessary for the symbioses because sexual progenies of most cnidarian species that host zooxanthellae (∼85%) acquire *Symbiodinium* cells from the immediate environment [7]. Corals then harbor the “foreign algae” intracellularly, and engage them as suppliers of photosynthetic products. The original deBary definition of symbiosis as a mutually beneficial association between the symbiont and host seems to apply to *Symbiodinium*-coral symbioses, and the translocated photosynthates provided by *Symbiodinium* confers prime nutritional advantage to the host. This must necessitate, at the very least, the transport of inorganic carbon, nitrogen and phosphorus sources through the host coral to the symbiont algae [8]. However, while there is considerable evidence for the translocation of photosynthetic products into the host corals, evidence regarding the benefits of the symbiosis for *Symbiodinium* is scanty.

Crystalline deposits in *Symbiodinium* cells had long been misidentified as oxalic acid [9], [10]. Recently, Clode *et al.* (2009) [11] showed the crystalline materials are uric acid by means of transmission electron microscopy (TEM) and energy-filtered TEM imaging analysis. In their study, addition of an inhibitor of a key enzyme for uric acid synthesis prevented new deposit formation. At the same time, this causes disappearance of existing deposits and is also thought to be due to utilization by the *Symbiodinium* cells [11]. It was concluded that a *de novo* purine pathway constitutes a major route for the assimilation of nitrogen in *Symbiodinium* cells. This provides new insight into the physiological strategies of *Symbiodinium*, which must survive in oligotrophic tropical environments, and suggests novel interactions of nutrient pathways between the symbionts and the host animals.

However, we believe that these deposits in *Symbiodinium* cells also function as eye-spots. In many marine animals, purines including uric acid have been identified as light reflectors [12]. In this paper, we report on light and transmission electron microscopy observations in cultured *Symbiodinium* motile cells that provide consistent evidence for the photoreceptive role of the crystalline deposits.

## Results

### Cell architecture of *Symbiodinium* in culture

**Figure 1:**
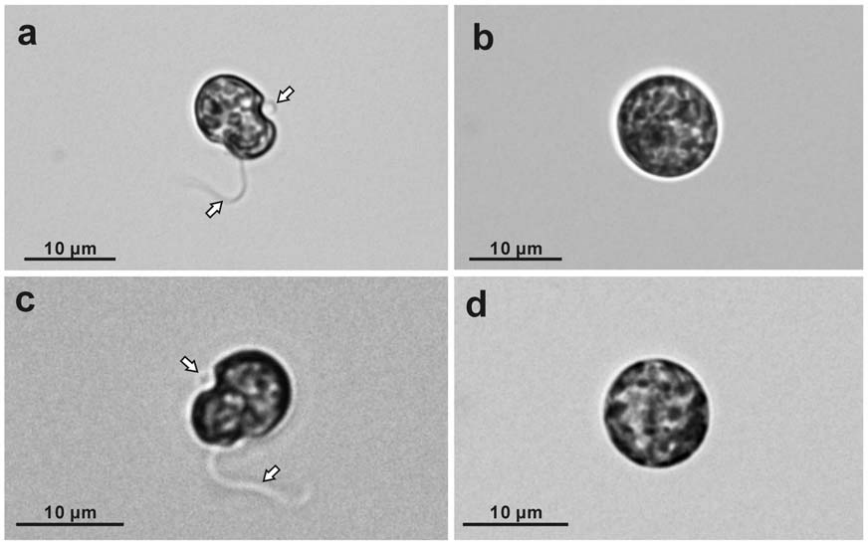
Light micrographs of cultured *Symbiodinium* cells. The upper row shows strain CS-156, and the lower row shows FKM0207. (a) and (c) Motile stage cells showing gymnodinioid morphology with transverse and longitudinal flagella (arrows) at 10:00. (b) and (d) Coccoid stage cells showing non-flagellated spherical shapes at 00:00.

**Figure 2:**
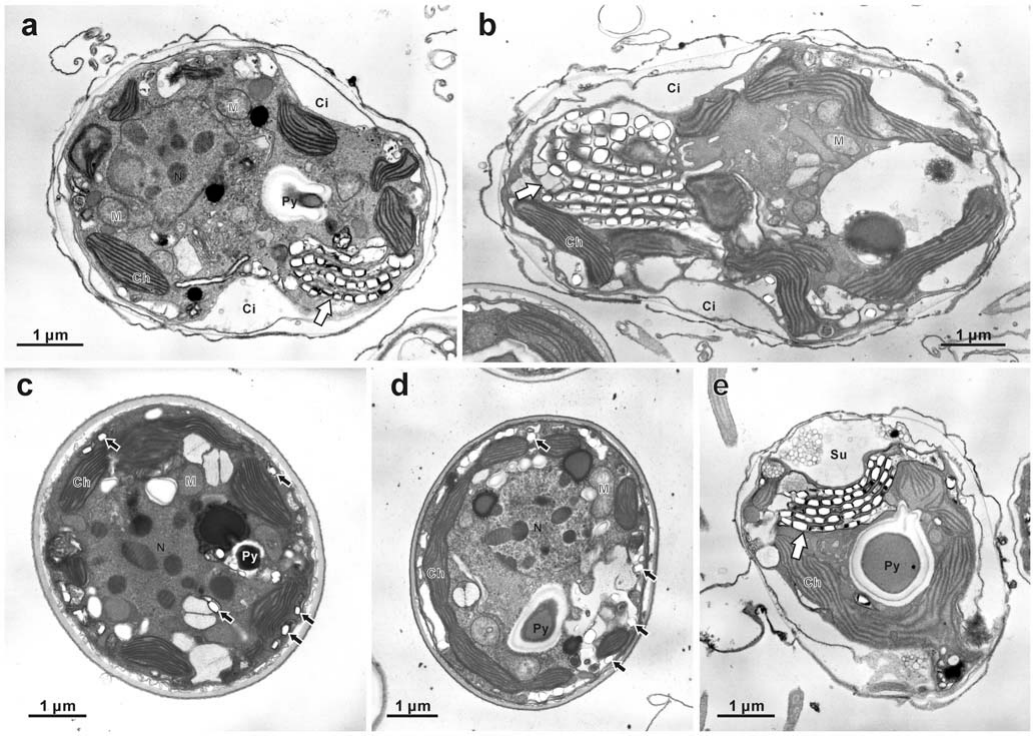
Transmission electron micrographs of cultured *Symbiodinium* cells. (a) Longitudinal section of strain CS-156 fixed at 10:00. (b) Longitudinal section of strain FKM0207 fixed at 10:00. In both (a) and (b) a cluster of crystalline deposit layers (white arrows) is located in the hypocone of the dinoflagellate cell. (c) Transverse section of CS-156 fixed at 00:00. (d) Transverse section of FKM0207 fixed at 00:00. These represent the thick-walled spherical morphology. Note that the crystalline deposits are not arranged in layers, but are sparsely scattered (black arrows). (e) Transverse section of CS-156 fixed at 10:00 showing the crystalline layers (a white arrow) underlying the sulcus region (Su) of the cell. Abbreviations: Dinokaryotic nucleus (N), mitochondrion (M), pyrenoid (Py), chloroplast (Ch), cingulum (Ci).

**Figure 3:**
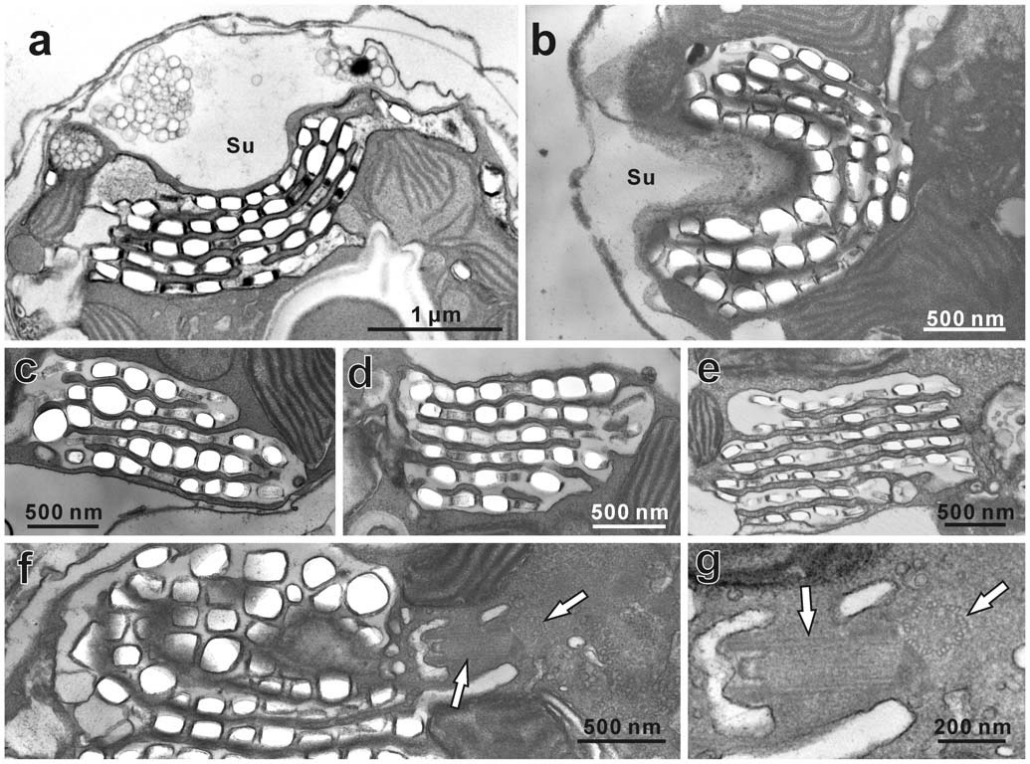
Transmission electron micrographs of crystalline layers found in the motile stage of *Symbiodinium*. Transmission electron micrographs showing close-ups of the crystalline layers found in the gymnodinioid motile stage in cultured *Symbiodinium* (CS-156 & FKM0207). (a) and (b) Arrangements of crystalline layers underlying the sulcus (Su). (c)–(e) Clusters with multiple layers of crystalline deposits. Five layers were most commonly found. (f) Two sections of a flagellar basal body can be seen (arrows) near the crystalline cluster. (g) Close-up of the flagellar basal body, showing circularly arranged triplet microtubules (right arrow).

Both cultured cell strains displayed characteristic gymnodinioid morphology with transverse and longitudinal flagella (Figures 1a, c: arrows) at 10:00 and spherical non-flagellated morphology (Figures 1b, d) at 00:00. In TEM observations, motile stage cells were covered with an undulating outermost cytoplasmic membrane and dinoflagellate- characteristic amphiesmal vesicles (Figures 2a, b), and changed drastically by 00:00 to thick-walled spherical morphologies (Figures 2c, d). In the daylight morphology (10:00) only, both strains possess a cluster of several rows of crystalline deposits near the cytoplasmic surface of the hypocone (the southern hemisphere of the dinoflagellate cell) (Figures 2a, b: arrows). The crystalline cluster is located near the sulcus (a groove where the longitudinal flagellum resides) (Figure 2e: arrows), and the cluster architecture was more or less the same in all cells: the rows followed the curvature of the sulcal groove (Figures 3a, b) with the flagellar basal body lying close to the cluster (Figures 3f, g: arrows). Each row consisted of a single membrane-bound layer containing aligned crystalline deposits. The clusters mostly consisted of five to seven rows (Figures 3c–e), but five rows being most common. Individual crystalline deposits were 80–130×150–250 nm in size, and the mean dimensions were 102×176 nm (*N* = 30). In contrast to the organized clusters of crystalline deposits in motile cells, the deposits were never arranged in rows or clusters, and were drastically decreased in number in the coccoid stage (Figures 2c, d: black arrows).

### Cell architecture of *Symbiodinium* in host animals

**Figure 4:**
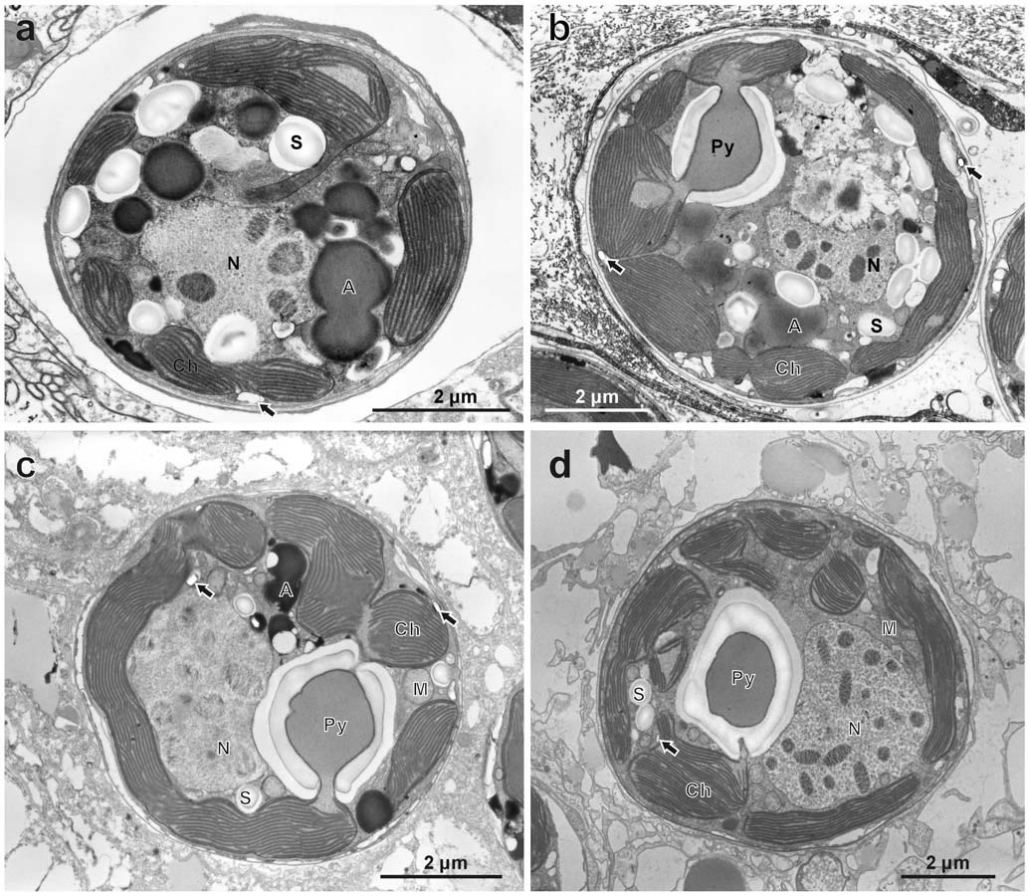
Transmission electron micrographs of *Symbiodinium* cells in animal hosts. *Symbiodinium* cells in (a) a sea anemone *Aiptasia* sp., (b) a giant clam *Tridacna crocea*, (c) a soft coral *Sinularia lochmodes*, and (d) a stony coral *Ctenactis echinata*. All samples were fixed at 10:00. There is no diurnal morphological change, and the cells are very similar to the coccoid stage cell in cultured strains. There are no crystalline layer clusters, while individual crystalline deposits can be scantily seen (arrows). Abbreviations: Dinokaryotic nucleus (N), mitochondrion (M), pyrenoid (Py), chloroplast (Ch), starch (S), accumulation body (A).

**Figure 5:**
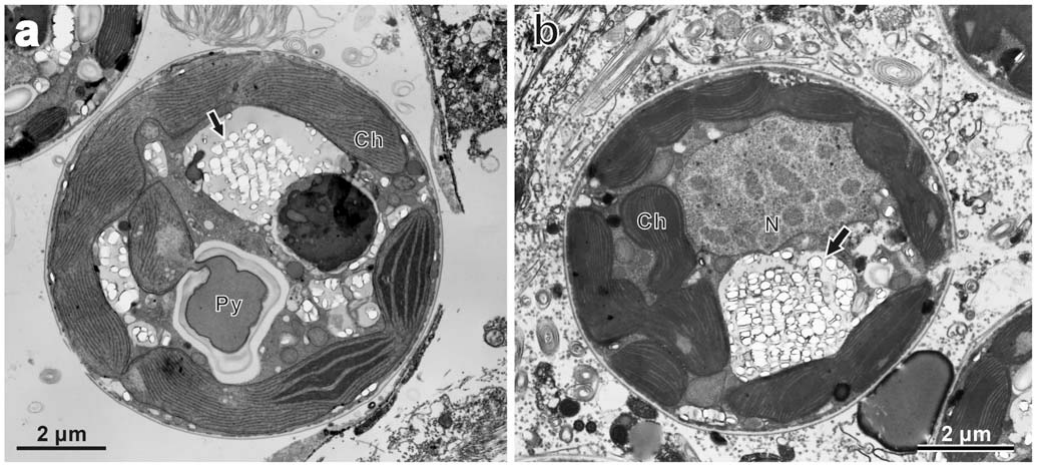
Transmission electron micrographs of *Symbiodinium* cells in the giant clam *Tridacna crocea*. (a) and (b) Noticeable crystalline clusters in random arrangements are found (arrows). The structure is far different from that found in cultured motile stage cells, and the crystalline deposits are tightly packed within vacuoles. Abbreviations: Dinokaryotic nucleus (N), pyrenoid (Py), chloroplast (Ch).

*Symbiodinium* in host animals usually maintain a coccoid morphology [13]. The symbionts within the four animals observed in this study did not exhibit diurnal morphological changes but maintained the coccoid morphology, similar to the coccoid stage of the cultured cells. All *Symbiodinium* cells in the host animals did not contain assemblies of crystalline deposits (Figure 4), with only a small number of individual deposits occasionally seen and randomly distributed (Figures 4: black arrows). In less than 10% of the total population of *Symbiodinium* associated with the clam (*N* = 300), irregularly arranged, tightly clustered crystalline deposit assemblages could be seen (Figures 5: black arrows).

### Light behavior around the eye-spot like region

**Figure 6:**
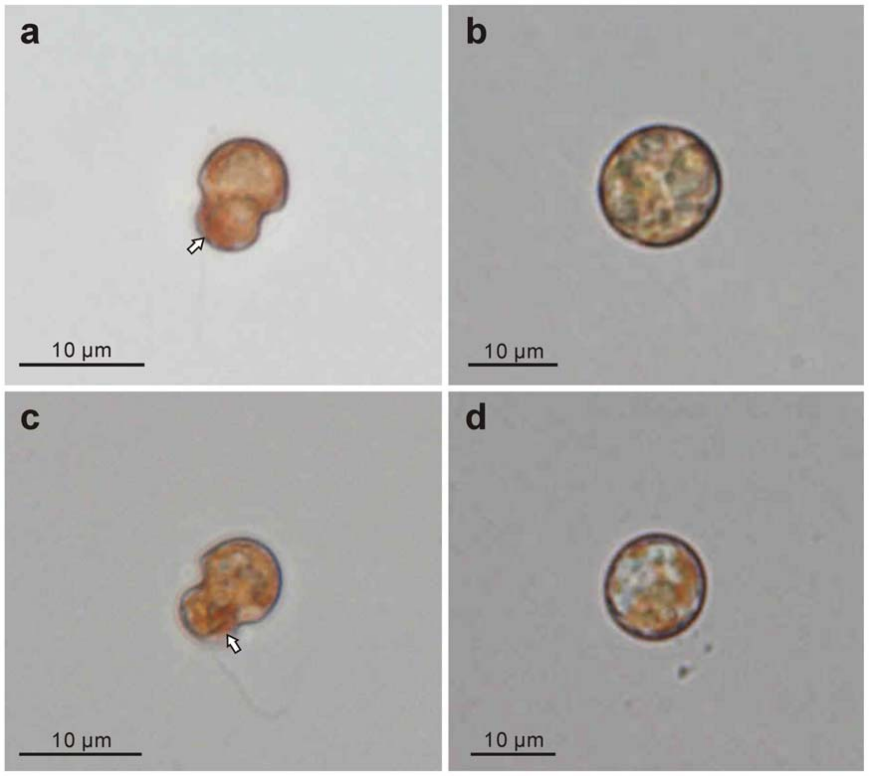
Color light micrographs of cultured *Symbiodinium* cells. The upper row shows strain CS-156, and the lower row shows FKM0207. (a) and (c) Motile stage cells with an orange-red body (arrows) near the sulcus. (b) and (d) Coccoid stage cells lacking an orange-red body.

**Figure 7:**
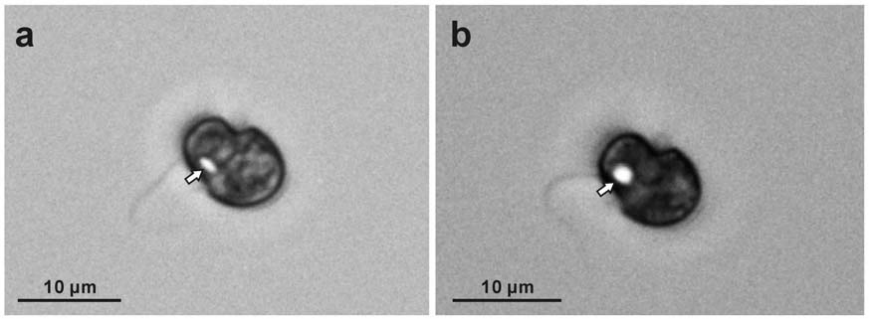
Cultured *Symbiodinium* motile cells viewed with differential interference contrast (DIC) microscope without the DIC analyzer. (a) CS-156. (b) FKM0207. Bright areas near the sulcus (arrows) indicate that strong light reflection and polarization may occur in this area corresponding to the location of the clusters as revealed by transmission electron microscopy.

**Figure 8:**
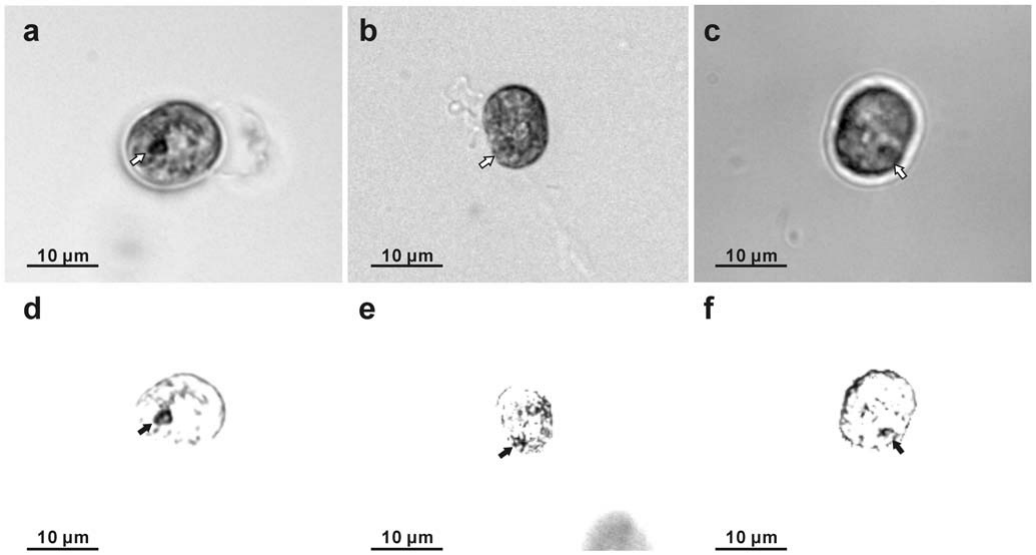
Light micrographs of cultured *Symbiodinium* motile cells under normal light and specific-wavelength light. (a)–(c) Normal light supply. The white arrows show the eye-spot like area. (d) Equivalent image field to (a) using a band-pass filter transmitting 365–400 nm (UV), attached to the microscope light port, (e) equivalent image field to (b) using a band-pass filter transmitting 410–492 nm (blue), and (f) equivalent image field to (c) using a band-pass filter transmitting 470–560 nm (green). Obvious black spots can be seen at the eye-spot like areas (black arrows), most visibly under UV.

In the color photomicrographs of cultured *Symbiodinium* (Figure 6), the motile cells showed an orange-red body near the sulcus (Figures 6a, c: arrows), which is absent in the coccoid stage (Figures 6b, d). In the DIC view, this area was characterized by the emission of brilliant light, which was enhanced when the DIC analyzer of the microscope was removed (Figure 7: arrows). This was probably due to polarization of transmitted light through the eye–spot like area; similar to polarization in some amorphous crystalline materials such as calcite. When the area was illuminated with the specific wavelength bands and visualized with ultra-sensitive CCD, the most obvious black spot at the eye-spot region appeared under UV radiation, followed by blue and then green illumination (Figure 8). This strongly indicates that the area deflects or absorbs the given light wavelengths, most strongly so in the case of UV radiation (Figures 8a, d).

## Discussion

The crystalline clusters observed in the motile gymnodinioid stage of *Symbiodinium* can be characterized as follows; (i) five rows of crystalline deposits forming an arc, situated near the sulcus region, (ii) each crystalline layer was 80–130 nm thick, (iii) the cluster could refract and polarize light in the manner of amorphous materials and noticeably deflected UV more than blue or green light, and (iv) they disappeared in the nocturnal coccoid stage.

In many marine animals, multilayer interference reflectors are found in the eyes (e.g. [12]). In most cases, each layer has a thickness of 1/4 of the wavelength of light, forming a “quarter-wavelength film” in which light reflected from the frontal surface of a transparent film interferes with the light reflected from the back surface of the film, and undergoes a 1/2 wavelength shift when passing twice through a 1/4 thickness film. The light reflection from the film can be maximized with interference resulting from both light passes. In the case of the *Symbiodinium* crystalline layer, each layer is 80–130 nm thick which is ideal for reflection of light with 320–520 nm wavelengths. Furthermore, the clusters are mostly composed of five layers, which are likely to be repeating units for light reflection. In multilayered reflector systems with 1/4 wavelength thickness in each reflecting layer, a reflectivity as high as that of a silver mirror (96.6%) can be achieved with as few as seven layers, if the refractive indices are well chosen [12]. Along with the remarkable discovering of uric acid in *Symbiodinium* crystalline deposits [11], there is evidence that uric acid layers probably function as reflector films. Purines, including uric acid, have been identified as the main constituents of reflecting structures in many marine animals [12], and interestingly, uric acid forms a reflective layer in the cuticle of scarab beetles [14].

**Figure 9:**
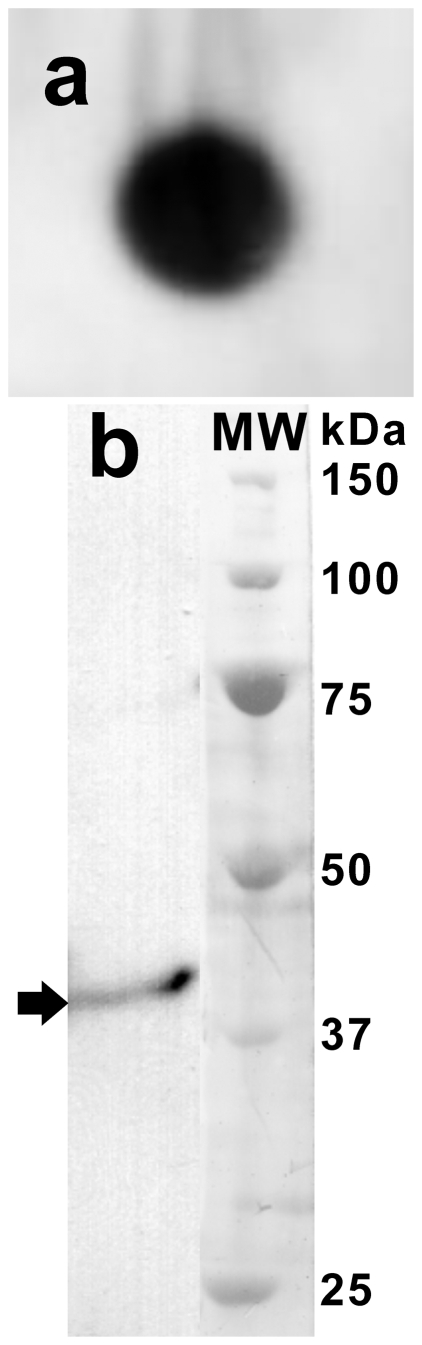
Northern- (a) and Western- (b) blot analyses showing a *Symbiodinium* (CS-156) opsin. (a) Northern dot blot analysis using a digoxigenin-labeled opsin cDNA probe for a dinoflagellate *Alexandrium tamarense*, showing a positive signal in the *Symbiodinium* total RNA. (b) Western blot analysis using an antibody against the partial *A. tamarense* opsin peptide, showing positive single band (arrow) in the *Symbiodinium* total protein. MW: Molecular weight marker.

If the crystalline clusters function as effective light reflectors, then a photoreceptor component should also be present. There is no published information regarding potential photoreceptors that could function with eye-spot structures in dinoflagellates. According to well-studied cases in the green alga *Chlamydomonas*, a visual pigment, rhodopsin, was identified [15], and it was shown that the rhodopsin particles were particularly abundant in the plasma membrane in the region of the eye-spot [16]. Therefore, we looked for such visual component in our *Symbiodinium* culture. Opsin, a protein element of rhodopsin, was putatively found in the cDNA library obtained from a dinoflagellate *Alexandrium tamarense* (Kobiyama *et al.*, in preparation). A digoxigenin (DIG) labeled cDNA probe against putative opsin RNA of *A. tamarense*, and an antibody against the artificially synthesized partial opsin peptide, were applied to the Northern- dot- blot analysis of *Symbiodinium* (CS-156) RNA and to the Western-blot analysis of the total protein from the same strain. Obvious signals were detected in both Northern and Western blot analyses (Figure 9), indicating that *Symbiodinium* actually possesses the visual pigment rhodopsin (structural information of the putative opsin in *A. tamarense* and *Symbiodinium* will appear in Kobiyama *et al.*, in preparation), although we need further evidence to show that the rhodopsin molecules are associated with the reflectors of the crystalline layers.

In dinoflagellates, four types of eye-spots are known: type A, an independent structure that is not membrane-bound; type B, an independent membrane-bound structure; type C, structure as part of a chloroplast; and type D, an elaborate eye-like structure called ocellus [17]. In addition to these four categories, Horiguchi & Pienaar (1994a) [18] found a structure in the dinoflagellate *Gymnodinium natalense* very similar to the one described in this study, and proposed a fifth category. They also noted that the crystal deposits were produced within the chloroplast in this species [19]. Although it was not specifically mentioned by authors in the texts, structures found in *Gymnodinium linucheae* [20], *Polarella glacialis* [21] and *Woloszynskia halophila* [22] could also be classified into this fifth category. A phylogeny based on 28S rRNA gene nests *G. linucheae* and *W. halophila* within the lineage of Suessiales, an order containing *Symbiodinium* [22], [23], and *P. glacialis* is closely related to this lineage based on an analysis of the 18S rRNA gene [21]. Therefore, this type of eye-spot might be a unique feature to this group. It should also be noted that *G. linucheae* has been now reclassified into the genus *Symbiodinium* [24].

The results of this study and other studies indicate that *Symbiodinium* motile stage cells possess true eye-spots composed of uric acid crystalline layers. The eye-spot structures were absent in the coccoid stage in both cultured cells and in cells residing within host animals, while a small number of individual crystalline deposits were sometimes seen in cells associated with host animals. The degradation of the crystal clusters during transformation from the motile stage to the coccoid stage indicates that they can be rapidly digested within the cell; and the uric acid can potentially be recycled as a nitrogen source as proposed by Clode *et al.* (2009) [11]. Nonetheless, it is interesting that free-living motile *Symbiodinium* may have a need for the eye-spot, while eye-spots would seem to have no apparent function in non-motile cells at night or inside hosts. Conversely, since controlling the diurnal morphological transformation pattern of *Symbiodinium* seems to require light cues [25], the lack of an eye-spot might lead to a disruption of such morphological transformations among *Symbiodinium* within host animals.

In our trials, using UV under epi-fluorescent microscopy, motile cells tend to move away from UV, corroborating a previous report by Horiguchi *et al.* (1999) [26] in which the dinoflagellates with an eye-spot show negative phototactic responses to UV, while the ones without an eye-spot show positive responses. If indeed the eye-spot is involved in controlling swimming behavior in the motile stage of *Symbiodinium*, the mechanisms may be important in initiating the coral-symbiont relationship. Sexual progenies of most cnidarian species that host zooxanthellae (∼85%) acquire *Symbiodinium* cells from the environment [7]. As such, there must be some mechanisms to attract *Symbiodinium* cells to the host, the most plausible mechanism proposed so far has been a chemosensory attraction (e.g. [27]). If eye-spot driven swimming behavior would be involved for such an attraction, it provides an interesting perspective on the manner of initial contact between *Symbiodinium* and animal hosts, as proposed in the “beacon hypothesis”, in which green fluorescent protein (GFP) in host may attract *Symbiodinium* [28].

In the giant clam *Tridacna crocea*, a small number of non-motile *Symbiodinium* cells possessed eye-spot like structures. This could be related to the “looseness” of the host-symbiont association in giant clams compared to other animal hosts. In giant clams, symbionts are harbored in special tube systems connected to the stomach [29]; whereas all cnidarian hosts harbor *Symbiodinium* intracellularly. *Symbiodinium* in giant clams may thus be more predisposed for transformation to the free living motile stage.

We propose that the uric acid deposits found in *Symbiodinium* cells by Clode *et al.* (2009) [11] may function as a component of eye-spots in motile stage cells. Further study of light-driven behavior in *Symbiodinium* is needed to elucidate the enigmatic ecology of free living *Symbiodinium* and the unknown mechanisms by which they engage in symbiotic relationships with corals, which is a fundamental ecological basis of coral reef ecosystems.

## Materials and Methods

### *Symbiodinium* cultures

Two culture strains of *Symbiodinium* were used for this study: strain CS-156, an isolate from a stony coral *Montipora verrucosa*, was purchased from the Commonwealth Scientific and Industrial Research Organization (CSIRO), and strain FKM0207 was isolated in our laboratory from the scleractinian coral *Fungia* cf. *fungites*. They were maintained in IMK medium (Wako, Tokyo, Japan) at 25°C and in 80–120 µmol photon m^−2^ sec^−1^ (12 h light and 12 h dark period). Four to seven days after inoculation into new medium, the cultures were used for the observations described in this report.

### Diurnal cycle of *Symbiodinium* morphology

*Symbiodinium* in culture show daily morphological changes between a flagellated gymnodinioid stage (motile stage) in daylight and a non-flagellated spherical stage (coccoid stage) at night [25], [30]–[34]. To confirm the diurnal cycle of the cultures, each strain was transferred to wells of a 24 well plate at a concentration of 1×10^4^ cells ml^−1^, and observed at 3 h intervals for 24 h under an inverted light microscope (IX71, Olympus, Tokyo, Japan). Maximum transformations to the motile stage and the coccoid stage were estimated to have occurred by 10:00 and 00:00 respectively.

### Transmission electron microscopy

At 10:00 and 00:00, 10 ml of cultures of both strains were fixed for 1 h in a solution of 2.5% glutaraldehyde, 0.15 M sucrose, and 2% tannic acid in 0.1 M cacodylate buffer (pH 7.4). The cells were then washed three times with 0.1 M cacodylate buffer followed by centrifugation (1,800×g, 15 min), and post-fixed with 2% OsO_4_ for 1.5 h at 4°C. After rinsing three times with the buffer, the pellet was then dehydrated through a graded ethanol series and n-butylglycidylether (Nisshin EM, Tokyo, Japan), and embedded in Spurr's resin. Sections were cut with a diamond knife using an ultra microtome (Ultracut UCT, Leica, Wien, Austria) and mounted on Cu grids. They were stained with uranyl acetate for 30 min and lead citrate for 5 min. Observations were carried out with a JEM-1011 transmission electron microscope (JEOL, Tokyo, Japan).

### Animal hosted *Symbiodinium* specimens

*Symbiodinium* hosted within a sea anemone *Aiptasia* sp., a giant clam *Tridacna crocea*, a soft coral *Sinularia lochmodes*, and a stony coral *Ctenactis echinata* were used for reference comparisons with the morphology of cultured cells. The animals, except *Aiptasia* sp., were collected from Aka Island, Okinawa, Japan, and temporarily maintained in an aquarium. *Aiptasia* sp. were kindly provided by Dr. T. Maruyama (Japan Agency for Marine Earth Science and Technology). At 10:00 and 00:00, whole bodies of *Aiptasia* sp. and tissue pieces of *T. crocea*, *S. lochmodes* and *C. echinata* were processed for TEM observations according to the protocol for cultured *Symbiodinium* cells, except that fixation times in glutaraldehyde solution were prolonged to 14 to 26 h. *S. lochmodes* and *C. echinata*, were decalcified in a solution of 4% EDTA-2Na+7% sucrose (pH 7.4) for 2 or 4 weeks prior to osmium fixation.

### Light microscopy

At 10:00 and 00:00, live CS-156 and FKM0207 cells were retrieved from the culture vessels, mounted on a glass slide without fixation, and observed with a differential interference contrast (DIC) microscope (BX-51, Olympus, Tokyo, Japan). Color microphotographs were taken with a 600 CL cooled digital camera (Penguin, Los Gatos, CA, USA) under non DIC lighting. A characteristic eye-spot like structure could be observed in the cells under the TEM. To know some light actions (deflection, diffraction, and absorption) around this area, DIC observation was performed: when the background was darken by adjusting a DIC prism, the eye-spot area readily appeared as a bright spot, and removal of the DIC analyzer enhanced the brightness of the spot, indicating polarization of the transmitted light (see Results for details). To determine the deflection or absorption properties under specific light wavelengths, band-pass filters (Nikon, Tokyo, Japan) transmitting 365–400 nm (UV), 410–492 nm (blue), 470–560 nm (green) were individually attached to the light port of the microscope, and the transmitted light through the cells was recorded using an ultra-sensitive CCD camera (Roper Scientific CoolSNAP ES, Tucson, AZ, USA).
